# AMPK α_1_ Activation Is Required for Stimulation of Glucose Uptake by Twitch Contraction, but Not by H_2_O_2_, in Mouse Skeletal Muscle

**DOI:** 10.1371/journal.pone.0002102

**Published:** 2008-05-07

**Authors:** Thomas E. Jensen, Peter Schjerling, Benoit Viollet, Jørgen F. P. Wojtaszewski, Erik A. Richter

**Affiliations:** 1 Molecular Physiology Group, Copenhagen Muscle Research Centre, Department of Exercise and Sport Sciences, Section of Human Physiology, University of Copenhagen, Copenhagen, Denmark; 2 Copenhagen Muscle Research Centre, Department of Molecular Muscle Biology, Rigshospitalet, Copenhagen, Denmark; 3 Medical Muscle Research Cluster, Molecular Muscle Biology, Department of Biomedical Sciences, University of Copenhagen, Copenhagen, Denmark; 4 Institut Cochin, Université Paris Descartes, CNRS (UMR 8104), Paris, France; 5 Inserm, U567, Paris, France; University of Giessen Lung Center, Germany

## Abstract

**Background:**

AMPK is a promising pharmacological target in relation to metabolic disorders partly due to its non-insulin dependent glucose uptake promoting role in skeletal muscle. Of the 2 catalytic α-AMPK isoforms, α_2_ AMPK is clearly required for stimulation of glucose transport into muscle by certain stimuli. In contrast, no clear function has yet been determined for α_1_ AMPK in skeletal muscle, possibly due to α-AMPK isoform signaling redundancy. By applying low-intensity twitch-contraction and H_2_O_2_ stimulation to activate α_1_ AMPK, but not α_2_ AMPK, in wildtype and α-AMPK transgenic mouse muscles, this study aimed to define conditions where α_1_ AMPK is required to increase muscle glucose uptake.

**Methodology/Principal Findings:**

Following stimulation with H_2_O_2_ (3 mM, 20 min) or twitch-contraction (0.1 ms pulse, 2 Hz, 2 min), signaling and 2-deoxyglucose uptake were measured in incubated soleus muscles from wildtype and muscle-specific kinase-dead AMPK (KD), α_1_ AMPK knockout or α_2_ AMPK knockout mice. H_2_O_2_ increased the activity of both α_1_ and α_2_ AMPK in addition to Akt phosphorylation, and H_2_O_2_-stimulated glucose uptake was not reduced in any of the AMPK transgenic mouse models compared with wild type. In contrast, twitch-contraction increased the activity of α_1_ AMPK, but not α_2_ AMPK activity nor Akt or AS160 phosphorylation. Glucose uptake was markedly lower in α_1_ AMPK knockout and KD AMPK muscles, but not in α_2_ AMPK knockout muscles, following twitch stimulation.

**Conclusions/Significance:**

These results provide strong genetic evidence that α_1_ AMPK, but not α_2_ AMPK, Akt or AS160, is necessary for regulation of twitch-contraction stimulated glucose uptake. To our knowledge, this is the first report to show a major and essential role of α_1_ AMPK in regulating a physiological endpoint in skeletal muscle. In contrast, AMPK is not essential for H_2_O_2_-stimulated muscle glucose uptake, as proposed by recent studies.

## Introduction

AMP activated protein kinase (AMPK) is emerging as an attractive target in both prophylaxis and treatment of metabolic disorders, including obesity and type 2 diabetes[1]. Key to its beneficial effects, AMPK promotes GLUT4 translocation and glucose uptake into skeletal muscle by a signaling cascade independent of the classical insulin-signaling cascade through PI3K-Akt[1].

Using AMPK signaling-deficient transgenic mouse models, various research groups have demonstrated that the skeletal muscle enriched catalytic α_2_ AMPK isoform is necessary to increase glucose uptake into skeletal muscle with certain stimuli, including 5-aminoimidazole-4-carboxamide ribonucleoside (AICAR), hypoxia and metabolic uncoupling[2]–[4]. Furthermore, both α_2_ AMPK and Akt signaling phosphorylate the Rab-GAP protein AS160, a probable regulator of GLUT4 translocation during contraction and insulin-stimulation[5], [6]. Together with studies showing α_1_ AMPK activation without an increase in glucose uptake during AICAR-stimulation in α_2_ AMPK knockout muscle (KO)[3], [7], [8], this suggests that α_1_ AMPK does not regulate glucose uptake.

Meanwhile, recent studies in incubated rat muscles have challenged the sovereignty of α_2_ AMPK in stimulating glucose uptake by demonstrating that the increase in glucose uptake elicited by hydrogen peroxide (H_2_O_2_) and low-intensity short-duration twitch-contraction is paralleled by an increase in α_1_ AMPK activity but not α_2_ AMPK activity[9], [10]. This paradigm was supported by another report in incubated mouse muscle, where H_2_O_2_-stimulated AMPK activation and glucose uptake coincided[11]. Reminiscent of Twitch/H_2_O_2_-stimulation, an α_1_ AMPK-exclusive activation profile was found in mouse soleus muscle stimulated with the sarcoplasmic reticulum (SR) Ca^2+^-releasing agent, caffeine[12]. Importantly, kinase-dead (KD) AMPK expression inhibited caffeine-stimulated glucose uptake[12]. However, the KD AMPK transgenic model reduces both α_1_ and α_2_ AMPK activity[13] and does not allow conclusions to be drawn about the relative importance of these to glucose uptake regulation. Therefore, whether increasing α_1_ AMPK activity, pharmacologically or by transcutaneous neuromuscular stimulation as suggested recently[14], can actually improve glucose homeostasis remains controversial as the studies above did not establish a causal relationship between α_1_ AMPK activation and glucose uptake.

Because both H2O2-stimulation and short-duration twitch-contraction appeared useful to activate α_1_ AMPK without activating α_2_ AMPK[9], [10], these stimuli were applied to incubated wildtype, α_1_ AMPK KO, α_2_ AMPK KO and kinase-dead (KD) AMPK muscles to answer whether alpha1 AMPK is necessary to increase glucose uptake in these conditions. Our data provides genetic evidence that α_1_ AMPK, but not α_2_ AMPK, Akt or AS160, is required for twitch-contraction stimulated glucose uptake. On the other hand, AMPK does not appear essential for H_2_O_2_-stimulated muscle glucose uptake, as proposed by recent studies.

## Results

### Initial characterization of twitch-contraction and H_2_O_2_-stimulated tension-development and signaling

**Figure 1:**
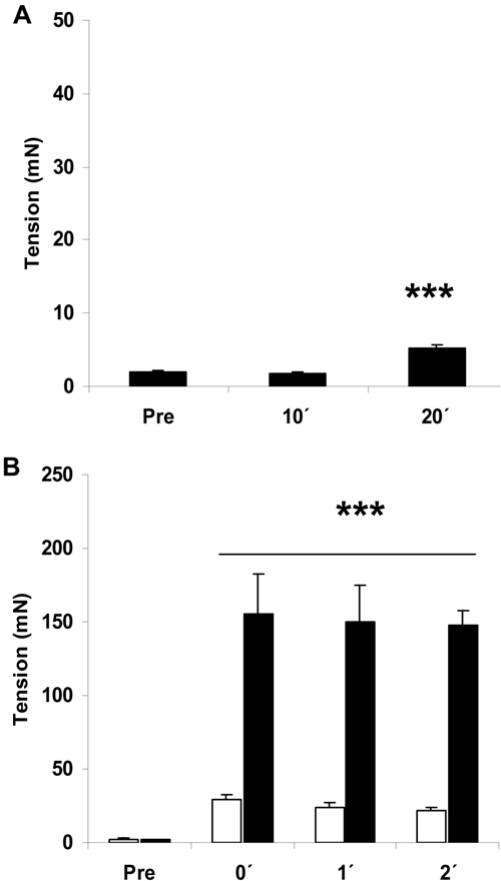
Tension-measurements. Tension-development during A) H2O2 stimulation (3 mM, 20 min) and B) twitch (white bars: 0.1 ms, 2 Hz, 2 min) and tetanic (black bars: 0.2 ms, 100 Hz, 1s/15s, 2 min) stimulation in mouse soleus muscles (n =  5–8). *** p<0.001 vs. pre.

H_2_O_2_-stimulation (3 mM) did not affect resting tension for the first 10 min, but slightly increased tension development from 10 to 20 min of stimulation (Figure 1A). Force-production during twitch-contraction reached an initial peak level around 30 mN and remained constant throughout the 2 min stimulation period (Figure 1B). Note that the peak force during the twitch-protocol is around 1/5 of the peak force observed during tetanic contraction of mouse soleus muscle (also shown in Figure 1B for comparison), emphasizing the difference between this and the more commonly used tetanic stimulation protocols [3], [15], [16].

**Figure 2:**
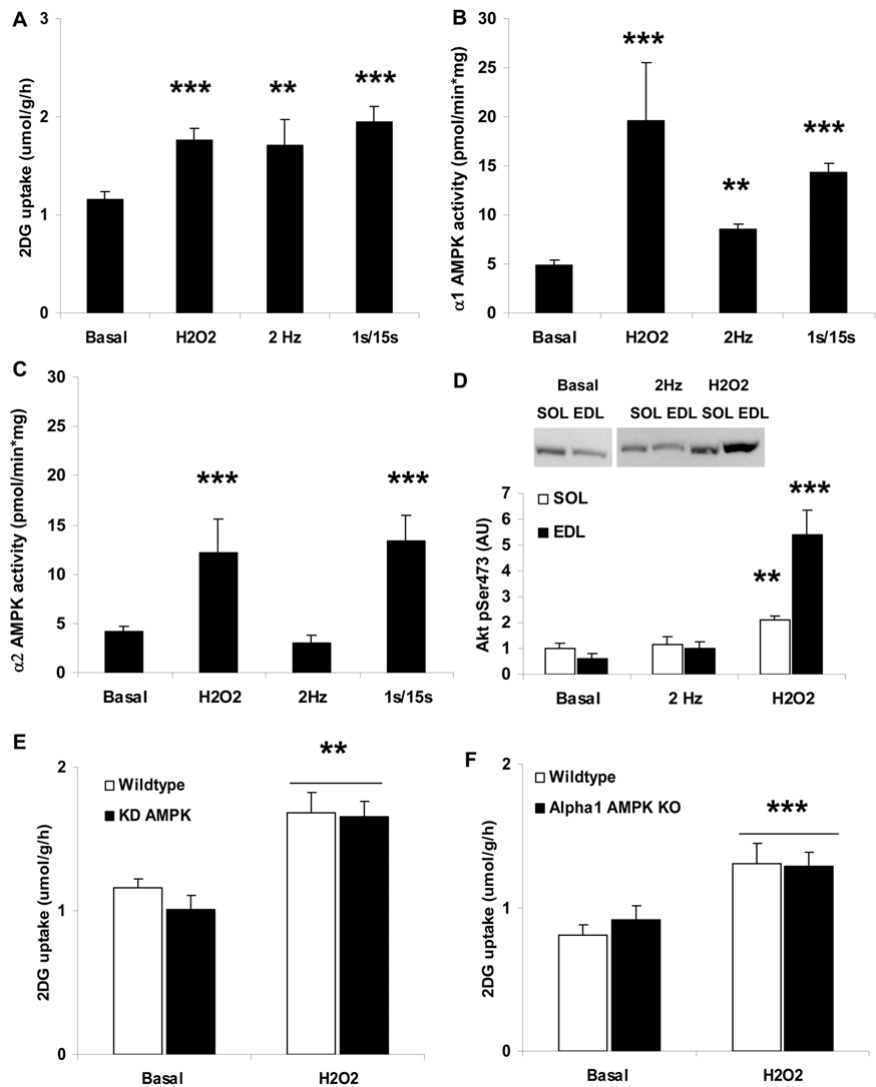
H_2_O_2_ and twitch-contraction stimulated 2-deoxyglucose (2DG) uptake and signaling in mouse soleus muscle. A) 2DG uptake in basal vs. H_2_O_2_ (3 mM, 20 min), twitch contraction (0.1 ms, 2 Hz, 2 min) or tetanic contraction (0.2 ms, 100 Hz, 1s/15s, 10 min)-stimulated muscles (n = 5–12). B) α_1_ AMPK and C) α_2_ AMPK activities in basal vs. H_2_O_2_ with same conditions as in panel A (n = 6–16). D) Akt Ser473 phosphorylation in basal, twitch-contracted and H_2_O_2_-stimulated soleus and EDL muscles (n = 6). E) H_2_O_2_-stimulated 2DG uptake in wildtype vs. kinase-dead (KD) AMPK muscles (n =  5–6) and F) wildtype vs. α_1_ AMPK KO muscles (n = 8). **/*** p<0.01/0.001 vs. basal.

Both 3 mM H_2_O_2_ for 20 min or twitch-contraction for 2 min increased glucose uptake to a similar extent as mild tetanic contraction for 10 min in incubated mouse soleus (Figure 2A). While twitch-contraction stimulated only α_1_ AMPK activity, H_2_O_2_ increased both α_1_ and α_2_ AMPK activities (Figure 2B and 2C). As has been demonstrated in rat epitroclearis muscle [10], prolonging 2Hz-stimulation to 5 min caused a significant increase in both α_1_ and α_2_ AMPK activity in mouse soleus muscle, making this time point useless to isolate α_1_ AMPK activation (α_1_ AMPK activity basal: 1.7 2 Hz, 5 min: 2.9, p = 0.008 α_2_ AMPK activity basal: 1.3 2 Hz, 5 min: 2.1, p = 0.013, n = 7). Previously, H_2_O_2_ has been shown to cause activation of many proteins in skeletal muscle, including Akt[17]. In our hands, H_2_O_2_, but not twitch-contraction, elicited a significantly ∼1 fold higher Akt phosphorylation in soleus (Figure 2D). To directly compare with the paper by Sandström and colleagues[11], we also measured H_2_O_2_-stimulated Akt phosphorylation in Extensor Digitorum Longus (EDL) muscles which was ∼4–5 fold higher compared with basal (Figure 2D). H_2_O_2_ stimulation of glucose uptake did not differ between wild type and either α_1_ AMPK KO (Figure 2E) or KD AMPK muscles (Figure 2F). Together, these results show that H_2_O_2_ stimulation of glucose uptake in muscle does not require AMPK catalytic activity and activates at least one other candidate glucose uptake promoting protein.

### Signaling following twitch contraction in AMPK transgenic mice

**Figure 3:**
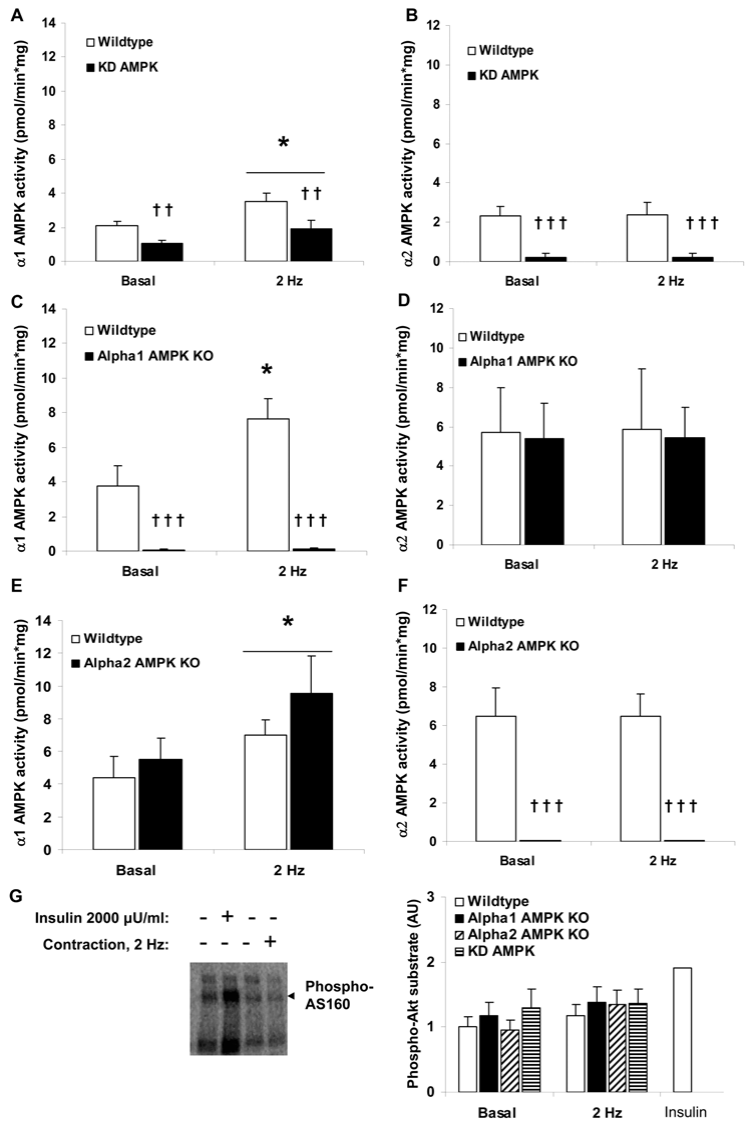
Twitch-contraction (0.1 ms, 2 Hz, 2 min) signaling in AMPK transgenic soleus muscles. A) α_1_ AMPK and B) α_2_ AMPK activities in wildtype vs. kinase-dead (KD) AMPK muscles (n = 7–8), C ) α_1_ AMPK and D) α_2_ AMPK activities in wildtype vs. α_1_ AMPK KO muscles (n = 7–10), E ) α_1_ AMPK and F) α_2_ AMPK activities E) and F) wildtype vs. α_2_ AMPK (n = 10). G) Basal and twitch-stimulated AS160 phosphorylation in wildtype vs. AMPK transgenic muscles (n = 6–9). As a positive control, insulin-stimulated (2000 µU/ml, 15 min) soleus was included (n = 1). * p<0.05 vs. basal. ††/††† p<0.01/0.001 genotype main effect.

**Figure 4:**
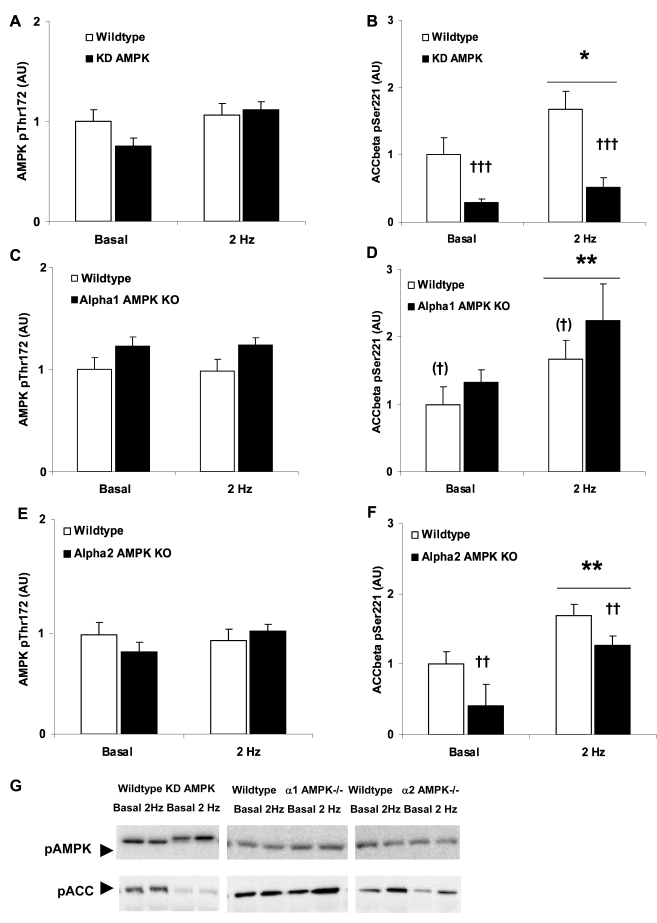
Twitch-stimulated (0.1 ms, 2 Hz, 2 min) AMPK and ACCβ phosphorylation in mouse soleus muscles. AMPK Thr172 phosphorylation in. A) wildtype vs. kinase-dead (KD) AMPK muscles (n = 7–8), C) wildtype vs. α_1_ AMPK KO muscles (n = 9–10) E) wildtype vs. α_2_ AMPK KO muscles (n = 10). ACCβ Ser221 phosphorylation in B) wildtype vs. kinase-dead (KD) AMPK muscles (n = 7–8), D) wildtype vs. α_1_ AMPK KO muscles (n = 9–10) F) wildtype vs. α_2_ AMPK KO muscles (n = 10). */** p<0.05/0.01 vs. basal. ††/††† p<0.01/0.001 genotype-effect. (†) indicates borderline significant genotype-effect, p = 0.06. G) Representative blots

Twitch-contraction increased α_1_ AMPK activity, but not α_2_ AMPK activity in wildtype muscles (Figure 3). α_1_ AMPK activity was ∼50% lower in KD AMPK muscles and absent in α_1_ AMPK KO muscles, while α_2_ AMPK activity was nearly absent in KD AMPK muscles and non-detectable in α_2_ AMPK KO (Figure 3A–3F). No compensatory increase in α_2_ AMPK activity was detected during twitch-contraction in the α_1_ AMPK KO muscles (Figure 3D) while the α_2_ AMPK KO muscles tended to display higher mean levels of basal and twitch-stimulated α_1_ AMPK activity (Figure 3E). Since neither α_2_ AMPK nor Akt (Figure 3D) appeared to be activated by the protocol currently employed, we asked whether AS160 phosphorylation, a point of convergence of α_2_ AMPK and Akt signaling to glucose uptake[6], was different from basal. AS160 phosphorylation was ∼100% higher with insulin-treatment but did not differ between twitch contraction-treated muscles and non-contracted muscles (Figure 3G). Thus, the increase in glucose uptake with twitch-contraction presumably cannot be explained by increased AS160 phosphorylation. No increases in AMPK Thr172 phosphorylation were detected in twitch contraction-stimulated wildtype or AMPK transgenic muscles despite significant increases in ACCβ Ser221 phosphorylation (Fig 4). This supports our previous observation in incubated mouse soleus muscles following sub-contraction threshold caffeine-stimulation, another α_1_ AMPK-specific stimulus, which likewise does not cause a significant increase in AMPK Thr172 phosphorylation, despite increasing ACCβ Ser221 phosphorylation [12].

### α_1_ AMPK is required for twitch-contraction stimulated glucose uptake

**Figure 5:**
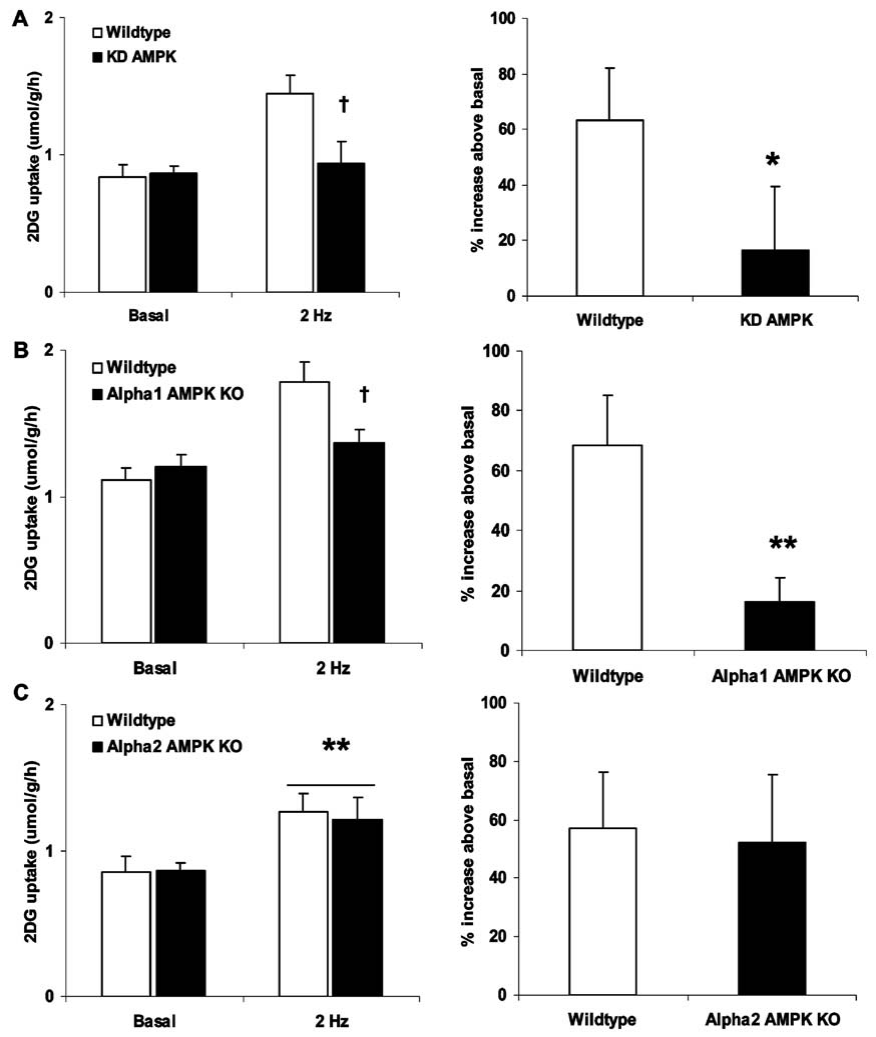
Twitch-contraction requires α_1_ AMPK to stimulate glucose uptake. Twitch contraction (0.1 ms, 2 Hz, 2 min) stimulated 2-deoxyglucose in mouse soleus muscles from either A) wildtype vs. kinase-dead AMPK muscles (n = 10–13) B) wildtype vs. α_1_ AMPK KO muscles (n = 16–17) C) wildtype vs. α_2_ AMPK KO muscles (n = 11–12). Absolute values are shown on the left and the corresponding percentage increase above basal for paired muscles is shown on the right. */** p<0.05/0.01 vs. wildtype or basal, † genotype x contraction p<0.05.

Twitch-contraction elicited a ∼60% increase in glucose uptake above basal in wildtype muscles (Figure 5). The corresponding increases were lower than 20% above basal in both the KD AMPK (Figure 5A) and α_1_ AMPK KO muscles (Figure 5B), but similar to wildtype in muscles lacking α_2_ AMPK (Figure 5C). This suggests that α_1_ AMPK is a required signaling component to glucose uptake stimulation during the low-intensity twitch-contraction regimen.

## Discussion

Stimulation of glucose uptake ex vivo by either H_2_O_2_ or twitch-contraction has been proposed to require α_1_ AMPK, based on correlations between α_1_ AMPK activity and glucose uptake in isolated rat epitroclearis muscle[9], [10]. This study provides the first clear genetic evidence for a causal link between α_1_ AMPK and glucose uptake-regulation following twitch-contractions. In contrast, H_2_O_2_-stimulated glucose uptake was not affected by reductions in AMPK activity.

To our knowledge, this is the first report of a metabolic regulatory role of α_1_ AMPK in skeletal muscle. Likely, the key to observing a major role of α_1_ AMPK in glucose uptake-regulation lies in the use of the low intensity, short duration contraction-regimen, which failed to increase α_2_ AMPK activity. In a previous study, glucose uptake ex vivo was only ∼20% lower in α_1_ AMPK KO soleus muscles compared to wildtype following intense tetanic ex vivo contraction (3). We speculate that intense tetanic contraction, like H_2_O_2_, activates other potential stimulators of glucose uptake, including Akt[18] and α_2_ AMPK[3], [13], which may have compensated for lack of α_1_ AMPK in that study.

A glucose uptake-promoting role of α_1_ AMPK is not unprecedented. In the rat liver epithelial clone9 cell line, α_1_ AMPK has thus been shown to be both necessary and sufficient to elicit an increased GLUT1-dependent glucose uptake, possibly due to movement out of detergent-resistant lipid rafts in the plasma membrane [19]–[24]. In skeletal muscle, GLUT4 is no doubt the major insulin and contraction-responsive glucose transporter isoform [25]–[27]. Still, GLUT4 KO mice retain some ability to increase glucose uptake in response to insulin and contraction into isolated muscles. Therefore, although speculative, it is possible that α_1_ AMPK targets GLUT1 or another non-GLUT4 glucose transporter expressed in skeletal muscle.

The twitch-contraction data support our previous findings, that caffeine-stimulated glucose uptake into mouse soleus muscle is AMPK-dependent[12]. Like twitch-contraction, caffeine-stimulated sarcoplasmic reticulum Ca^2+^-release activates α_1_ AMPK and phospho-ACC, but not α_2_ AMPK or phospho-AMPK, suggesting that the two stimuli are working through the same Ca^2+^-dependent pathway, proposed to involve the putative muscle-AMPK kinase, CaMKK[12]. A possible link between Ca^2+^-release by caffeine and AMPK activation is supported by another recent report using perfused rat hindlimb [28]. However, as discussed in a previous paper [12], any change in Ca^2+^ will perturb energy balance and potentially increase AMP/ATP-ratio. Whether the AMPK activation by caffeine is indeed Ca^2+^ dependent or can be explained by low-level changes in nucleotides [12] needs careful examination. Regardless, both α_1_ and α_2_ AMPK activities increase during in vivo exercise in mice[3] suggesting a physiological signaling role for both isoforms. Also, α_1_ AMPK expression and activity is increased compared to wildtype in both the whole-body α_2_ AMPK knockout mice[3] and in mice with muscle-specific deletion of the muscle-AMPK kinase, LKB1[8], further suggesting a compensatory role of α_1_ AMPK. However, as discussed previously[12], although α_1_ AMPK amount increases with exercise training[29], its activation during exercise is rarely observed in exercising human quadriceps-muscle. Still, it is conceivable that human muscles other than the principal biopsy-sampling muscle, quadriceps, more readily activate α_1_ AMPK during contraction. An exercise-study taking biopsies from the human soleus muscle, a method experimentally feasible [30], will be needed to resolve this question.

α_1_ AMPK activity was only reduced by ∼50% in the KD AMPK muscles yet glucose uptake stimulation by twitch-contraction was largely prevented. However, it has been proposed that residual α_1_ AMPK activity in the KD AMPK mice and a similar model, the muscle-specific α_2_i mice, may largely stem from non-muscle tissue present in muscle[2], [4], [13]. Therefore, the partial reduction in α_1_ AMPK activity could reflect a near-total reduction in muscle α_1_ AMPK activity. Other possibilities are that KD AMPK expression lowers α_1_ AMPK activity below a threshold required to increase glucose uptake or that KD AMPK expression affects α_1_ AMPK location and/or function by degrading α_2_ AMPK and AMPK-regulatory subunits. A similar paradox is that AICAR-stimulated glucose uptake is abolished in α_2_ AMPK KO muscles, despite α_1_ AMPK activity increasing[3]. Together with the current data, one straight-forward interpretation is that α_1_ AMPK provides a necessary, but not sufficient, signal to increase glucose uptake.

H_2_O_2_, working through AMPK, has been proposed to be involved in contraction-stimulated glucose uptake[9], [11]. In other studies, H_2_O_2_ concentrations below ∼1 mM do not seem to stimulate glucose uptake[31], [32], but may inhibit insulin-stimulated glucose uptake[31], while higher H_2_O_2_ concentrations increase glucose uptake in various muscle model-systems[9], [11]. The physiological range for H_2_O_2_ has been reported to be in the range of ∼30–150 µM[33]. Therefore, using mM concentrations to increase glucose uptake is likely not physiologically meaningful and should not be interpreted in this context. Apart from AMPK, H_2_O_2_ concentrations ranging from 60 µM-3 mM activate many signaling proteins, including MAPKs[31], [34], insulin receptor, IRS-1 and Akt[17], [31][and present study]. Possible activation mechanisms include reversible inactivation of phosphatases or activation of kinases by cysteine oxidation[35] and increased intracellular Ca^2+^ due to increased Ca^2+^-leak[36] and/or decreased uptake into the SR[33]. Furthermore, our finding of a minor increase in resting tension during H_2_O_2_ incubation, likely due to increased intracellular Ca^2+^ and/or increased myofibrillar Ca^2+^-sensitivity[33], opens up the possibility of metabolism and/or stretch-dependent signaling mechanisms. Based on the above and our studies, we strongly question the specificity and therefore usefulness of H_2_O_2_ in characterizing the relative contribution of AMPK or other signaling-molecules to contraction-stimulated glucose uptake. Furthermore, while this study was under revision, it was demonstrated in isolated rat EDL that 600 µM H_2_O_2_ potently activated Akt, but not AMPK, and glucose uptake in a wortmannin-sensitive manner [37], lending further support to the non-AMPK dependence of H_2_O_2_-stimulated glucose uptake.

Due to breeding difficulties, the α_1_ AMPK KO strain is currently being backcrossed from the C57BL/6 to the 129/SV mouse strain, and was 4^th^ to 5^th^ generation backcrossed at the time of experimentation (4^th^ generation theoretically 93.75% 129/SV and 6.25% C57BL/6). Genetic background is known to strongly influence effect sizes of various measuring endpoints (see [12] for discussion and references). However, our finding of reduced glucose uptake in both the C57BL/6 KD AMPK mice and the predominantly 129/SV α_1_ AMPK KO mice, suggests that the dependence of twitch-stimulated glucose uptake on α_1_ AMPK is not strain-specific.

The band detected by the PAS antibody in mouse soleus muscle is AS160 [38]. Unlike recent studies showing that signaling through AS160 is required for glucose uptake stimulation in adipocytes and muscle[39], twitch-contraction defines a condition where AS160 phosphorylation apparently does not correlate with glucose uptake. This suggests that α_1_ AMPK, unlike Akt and α_2_ AMPK[5], [6], is not an AS160-kinase. However, AS160 phosphorylation evaluated with the PAS antibody was recently shown to reflect mostly Thr642 phosphorylation[40]. Therefore, it remains possible that twitch-contraction targets other of the 8 identified AS160 phosphorylation sites[40]. Another possibility is that the change in AS160 phosphorylation measured with PAS following twitch-contraction was below the detection limit.

In conclusion, the present study 1) demonstrates that α_1_ AMPK activation is necessary to increase muscle glucose uptake following twitch-contraction and 2) provides the first genetic evidence for an essential role of α_1_ AMPK in contraction-stimulated skeletal muscle glucose uptake. In contrast, H_2_O_2_ is not an AMPK-specific stimulus.

## Materials and Methods

### Animals

Generation of the α_1_ and α_2_ AMPK whole-body KO as well as the muscle-specific KD α_2_ AMPK mice has been described previously [3], [4], [41]. The α_1_ AMPK KO mice were mixed males and females from 4^th^ and 5^th^ generation backcross of C57Bl/6 onto 129/SV-background. The α_2_ AMPK KO mice were fully backcrossed (>10^th^ generation) C57Bl/6 males. The KD AMPK mice were mixed males and females from 6^th^ and 7^th^ generation backcross of C57Bl/6 onto C57Bl/6. In all cases, sex and age-matched wild type littermates were used as controls. Female C57Bl/6 mice were used for all other experiments in this study. All mice were 12–18 wks old, when experiments were performed. Experiments were approved by the Danish Animal Experimental Inspectorate and complied with the “European Convention for the Protection of Vertebrate Animals Used for Experiments and Other Scientific Purposes”.

### Muscle incubation

Soleus or EDL muscles were obtained from fed anesthetized mice (6 mg of pentobarbital 100 g^−1^ body weight) and suspended at resting tension (4–5 mN) in incubation-chambers (Multi Myograph system; Danish Myo-Technology, Aarhus, DK) in Krebs-Ringer-Henseleit buffer (KRH) supplemented with 2 mM pyruvate and 8 mM mannitol at 30°C [3]. Muscles were preincubated for 1 h before measuring glucose uptake or signaling. H_2_O_2_ (3 mM) in KRH-buffer was added for the last 20 min. Muscle contraction was elicited by electrical stimulation with either 0.1 ms pulses at 2 Hz (∼50 V) for the last 2 or 5 min (termed twitch-contraction throughout this paper) or 1 s trains (0.2 ms, 100 Hz) every 15s for the last 10 min. Force development was measured during all incubations by a force transducer hooked to one end of the muscles.

### 2-Deoxyglucose Uptake

Following stimulation, glucose uptake was evaluated by measuring accumulation of ^3^H-labelled 2-deoxyglucose for 10 min, with ^14^C Mannitol as extracellular space marker[3].

### Muscle Analyses

Basal or stimulated muscles were quick-frozen by immersion in liquid nitrogen and processed into lysates[13]. Lysates were subjected to standard immunoblotting techniques[13], using the following phospho-specific antibodies: AMPK Thr172 (Cell Signaling Technology, MA), ACCβ Ser221 (Upstate Biotechnologies, MA), Akt Ser473 (Cell Signaling Technology), and phospho-Akt substrate motif R*X*R*XX*S/T (PAS) (Cell Signaling Technology) recognizing phospho-AS160[6].

### α_1_ and α_2_ AMPK Activity

Isoform-specific α-AMPK activity was measured *in vitro* in sequential immunoprecipitations from 200 µg of muscle lysate protein using anti-α_1_ and anti-α_2_ antibodies using AMARA peptide[13].

### Statistical analysis

Results are mean±SEM. Statistical testing was performed using unpaired t-tests or ANOVA with Tukey's honest significant difference post hoc test. An underlined symbol denotes bars that meet the criteria represented by that symbol. Statistical evaluation was performed using SPSS 15.0 for Windows. The significance level was set at α = 0.05.
